# Prognostic nutritional index as a prognostic biomarker for survival in digestive system carcinomas

**DOI:** 10.18632/oncotarget.13472

**Published:** 2016-11-12

**Authors:** Yang Zhao, Peng Xu, Huafeng Kang, Shuai Lin, Meng Wang, Pengtao Yang, Cong Dai, Xinghan Liu, Kang Liu, Yi Zheng, Zhijun Dai

**Affiliations:** ^1^ Department of Oncology, The Second Affiliated Hospital of Xi'an Jiaotong University, Xi'an, Shaanxi Province, PR China

**Keywords:** prognostic nutritional index, digestive system carcinomas, prognosis, meta-analysis

## Abstract

The prognostic nutritional index (PNI) has been reported to correlate with the prognosis in patients with various malignancies. We performed a meta-analysis to determine the predictive potential of PNI in digestive system cancers. Twenty-three studies with a total of 7,384 patients suffering from digestive system carcinomas were involved in this meta-analysis. A lower PNI was significantly associated with the shorter overall survival (OS) [Hazard Ratio (HR) 1.83, 95% Confidence Interval (CI) 1.62–2.07], the poorer disease-free survival (DFS) (HR 1.85, 95% CI 1.19–2.89), and the higher rate of post-operative complications (HR 2.31, 95% CI 1.63–3.28). In conclusion, PNI was allowed to function as an efficient indicator for the prognosis of patients with digestive system carcinomas.

## INTRODUCTION

According to the International Classification of Diseases for Oncology, digestive system carcinomas include esophageal, gastric, hepatocellular, colorectal and pancreatic carcinomas [childhood and adolescent carcinomas are classified according to the International Classification of Childhood Cancer (ICCC)] [1]. Despite the great improvement of early diagnosis, surgical skills, and multidisciplinary treatment in patients with digestive system carcinomas, digestive system carcinomas have been a major medical problem with high morbidity, mortality, and economic burden in a variety of human cancers [2–4]. Surgery remains the cornerstone for solid tumour treatment in patients fitting to operate. However, a huge number of patients (50%–70%) present post-operative complications or short-time relapse after surgery [5–7]. A bunch of groups demonstrated that the preoperative conditions of patients with digestive system carcinomas, especially for the nutrition and immune status, are associated with both the disease prognosis and the long-term post-operative outcomes of patients [8–17].

Several nutritional and immunological indicators, such as PNI, platelet-to-lymphocyte ratio (PLR), neutrophil-to-lymphocyte ratio (NLR), and the Glasgow prognostic score (GPS), have effeciently functioned as the assessable factors for uncovering the prognosis of cancers patients and for procuring the optimal therapy [9–12]. The nutritional index was initially introduced to work as a predictor for surgical risk by Buzby *et al*. in 1980 [15], which was further confirmed by Onodera *et al*. in 1984 [13]. Recent studies showed that low PNI was characterized as an independent prognostic factor for post-operative complications and short-term survival in various digestive system carcinomas [5, 16, 17]. Moreover, PNI has been extensively assessed in the clinical practice owing to its efficiency, simplicity, and convenience for predicting the pre-operative status and the surgical risk for gastrointestinal malignancy patients [13–15]. PNI is calculated by two obtainable values: the serum albumin concentration and the total lymphocyte count in the peripheral blood. However, many of these studies were conducted with small sample size and lacking the statistical power to reach convincing conclusions. With the goal of providing more powerful evidence to confirm the independent prognostic role of PNI in digestive system carcinomas, we conducted a meta-analysis of published studies regarding the association between the PNI and the prognosis of digestive system carcinomas.

## RESULTS

### Included studies and characteristics

**Figure 1:**
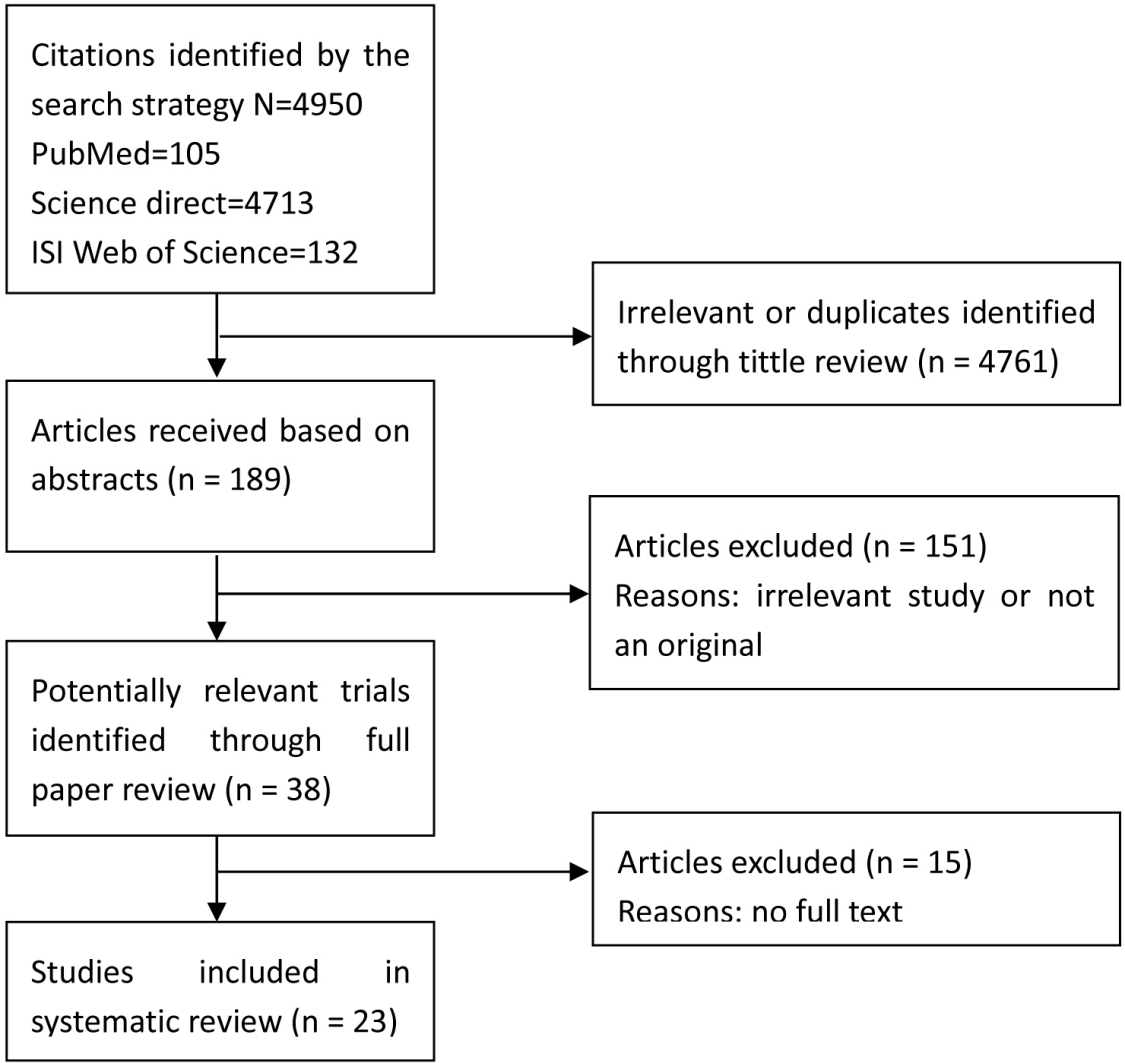
Flow diagram of the meta-analysis

As shown in Figure 1, we initially selected a total of 4,950 articles. After excluding 4761 articles due to the irrelevance to the HRs/odds ratios (ORs) as well as the duplicate articles, the abstracts of 189 studies were reviewed. Of 189 studies, 38 articles were eligible for further checking. 15 out of 38 articles were excluded owing to providing no access to the full text. Thus, we ultimately included 23 studies in our meta-analysis [4–7, 9, 16–33]. We assessed the quality of all included eligible articles according to the Newcastle-Ottawa scale, in which one article got a maximum of 9 stars, and determined that 56.5% of the studies (13 out of 23) had more than 6 stars. The main characteristics of the eligible articles were summarized in Table 1. The publication date of the articles covered a time span from 1981 to 2015. Most of the eligible studies were published in the past 5 years.

**Table 1:** Characteristics of studies included in the meta-analysis

Author	Date	Tumor Type	Region	Sample size	Age of analyzed population (years)	Clinical stage of tumour	Follow-up (months)(median and range)	Treatment	Cut-off value	Outcome	Survival analysis	Quality Score (NOS)
Nozoe T	2010	Gastric carcinoma	Japan	248	(27–89) PNI H: 64.4 ± 11.1PNI L: 70.1 ± 8.6	TNM I–IV(Japanese Classification of Gastric Carcinoma)	(1.7–110)	surgery	49.7	OS	multivariate analysis	5
Nozoe T	2012	Colorectal carcinoma	Japan	219	(24–90)PNI H: 69.8 ± 11.6 PNI L:74.5 ± 8.8	TNM I–IV(the 7th edition of the International Union against Cancer TNM Classification of Malignant Tumors)	(2–86)	surgery	40	OS	multivariate analysis	4
Pinato DJ	2012	Hepatocellular carcinoma	Japan	112	65 (20–83)	A-D (BCLC stage)	NA	surgery	45	OS	univariate analysis multivariate analysis	6
Watanabe M	2012	Gastric carcinoma	Japan	99	PNI H:79.2 ± 3.6 PNI L:80.3 ± 4.2	TNM I–IV (3rd English edition Japanese classification of gastric carcinoma)	(1–60)	surgery	44.7	OS	univariate analysis multivariate analysis	5
Migita K	2013	Gastric carcinoma	Japan	548	67 (24–89)	TNM I–IV (7th edition of the American Joint Committee on Cancer TNM classification system)	45.1	surgery	48	OS	univariate analysis multivariate analysis	7
Mohri Y	2013	Colorectal carcinoma	Japan	365	NA	TNM I–IV (the 7th edition of the International Union against Cancer TNM Classification of Malignant Tumors)	NA	surgery	45	OS and Post-operative complications	univariate analysis multivariate analysis	6
Maeda K	2014	Colorectal carcinoma	Japan	100	60.4 ± 10.6 (39–87)	TNM IV (the 7th edition of the International Union against Cancer TNM Classification of Malignant Tumors)	NA	surgery: Palliative Resection	40	OS	univariate analysis multivariate analysis	5
Feng JF	2014	Esophageal squamous cell carcinoma	China	375	59.1 ± 7.8 (36–80)	NA	(3–25)	surgery	42	CSS	univariate analysis multivariate analysis	4
Ishizuka M	2014	Gastric carcinoma	Japan	154	PNI H:64 ± 12 PNI L:69 ± 12	TNM I-IV and Type 0–5 (2nd English edition OF the Japanese classification of gastric carcinoma)	NA	surgery	45	OS	univariate analysis multivariate analysis	7
Jiang N	2014	Gastric carcinoma	China	386	60 (20–80)	TNM I–IV (7th edition of the American Joint Committee on Cancer TNM classification system)	39 (1–103)	surgery	46	OS and Post-operative complications	univariate analysis multivariate analysis	7
Ikeya T	2015	Colorectal carcinoma	Japan	80	63 (36–80)	TNM I–IV (the 7th edition of the International Union against Cancer TNM Classification of Malignant Tumors)	30.5	chemotherapy	44.5	OS	univariate analysis multivariate analysis	6
Okamura Y	2015	Hepatocellular carcinoma	Japan	341	69.5 (30–86)	TNM I–IV (the 7th edition of the International Union against Cancer TNM Classification of Malignant Tumors)	NA	surgery: Hepatectomy	48.5	OS and DFS	multivariate analysis	5
Chan AW	2015	Hepatocellular carcinoma	China	324	56.8 ± 10.9 PNI H:55.9 ± 10.7PNI L:59.3 ± 11.0	0-A (BCLC stage)	NA	surgery	45	OS and DFS	univariate analysis multivariate analysis	6
Sun KY	2015	Gastric carcinoma	China	632	57 (19–89)	TNM I–IV (7th edition of the American Joint Committee on Cancer TNM classification system)	55.75 (0.8–186)	gastrectomy and chemotherapy	48	OS	univariate analysis multivariate analysis	5
Shibutani M	2015	Colorectal carcinoma	Japan	218	69 (42–86)	TNM I–IV (the 7th edition of the International Union against Cancer TNM Classification of Malignant Tumors)	NA	surgery	45	OS	univariate analysis multivariate analysis	6
Proctor MJ	2011	Colorectal carcinoma	Scotland	374	NA	A–D (Dukes stage)	51 (18–115)	NA	45	OS and CSS	multivariate analysis	7
Kinoshita A	2012	Hepatocellular carcinoma	Japan	150	72 (43–91)	TNM I–IV (TNM classification of the liver Cancer Study group of Japan)	18 (1–80)	Surgical resection performed in 9 patients	45	OS	univariate analysis multivariate analysis	6
Nozoe T	2002	Oesophageal carcinoma	Japan	258	63.6 ± 9.5	TNM I–IV (TNM stage of the Japanese Society for esophageal Diseases)	32 (1–110)	surgery:Oesophageal resection and reconstruction	46	OS and Post-operative complications	Stepwise logistic regression analysis	4
Kanda M	2011	Pancreatic carcinoma	Japan	268	63 (35–83	TNM I–IV (the 7th edition of the International Union against Cancer staging Classification of Pancreatic cancer)	NA	surgery:Pancreatectomy	45	OS	univariate analysis multivariate analysis	6
Wang DS	2012	Pancreatic carcinoma	China	177	NA	TNM I-IV (the 7th edition of TNM staging of AJCC)	31.33 (10.80–59.70)	Macroscopically radical surgery performed in 31 patients; bypass or stenting surgery performed in 49 patients	45	OS	univariate analysis multivariate analysis	5
Geng Y	2015	Pancreatic carcinoma	China	321	PNI H:60.4 ± 11.0 PNI L:62.2 ± 10.2	TNM III-IV (6th edition of the International Union Against Cancer)	NA	chemotherapy	47.3	OS	univariate analysis multivariate Cox regression analyses	6
Eo WK	2015	Gastric carcinoma	Korea	314	59 (25–92)	TNM I-III (the 7th edition of TNM staging of AJCC)	36.5 (1.7–91.4)	surgery:curative surgical resection	47.3	OS and DFS	univariate analysis multivariate analysis	6
Jian-Hui C	2015	Colorectal carcinoma	China	1321	57.5 (18-91)	TNM I–IV (7th edition of the American Joint Committee on Cancer TNM classifcation system)A-D(Dukes stage)	NA	surgery	45	OS	univariate analysis multivariate analysis	6

A total of 7,384 participants were enrolled with the sample sizes of the individual studies ranging from 80 to 1321 (mean size: 321). 22 of 23 studies originated from Asian countries (14 from Japan, 7 from China and 1 from Korea), and one from Scotland. Five patterns of carcinomas were analysed in this systematic review: gastric cancer (7 studies), colorectal cancer (7 studies), hepatocellular carcinoma (4 studies), pancreatic cancer (3 studies), and esophageal carcinoma (2 studies). Seventeen studies investigated the prognostic significance of PNI with respect to post-operative outcomes, while 6 studies did not disclose whether patients had received surgery or what percentage of patients received surgery. The cut-off value of the PNI ranged from 40 to 49.7 with 43.5% of the studies (10 of 23) having a cut-off value set at 45. In accordance with the respective cut-off values, all studies divided patients into two groups: patients with low-PNI and high-PNI group. OS, DFS/PFS, cancer-specific survival (CSS), and post-operative complications were the main variances in the enrolled studies. There were 30 HRs/ORs extracted from 23 studies, including 22 for OS, 3 for DFS, 2 for CSS, and 3 for post-operative complications. Most of HRs/ORs and 95% CIs of the estimates could be directly obtained from 17 studies, while the small part of the estimates were indirectly calculated using the HRs and *P* values or the relative data from other 6 studies.

### Relationship between PNI and OS

**Figure 2:**
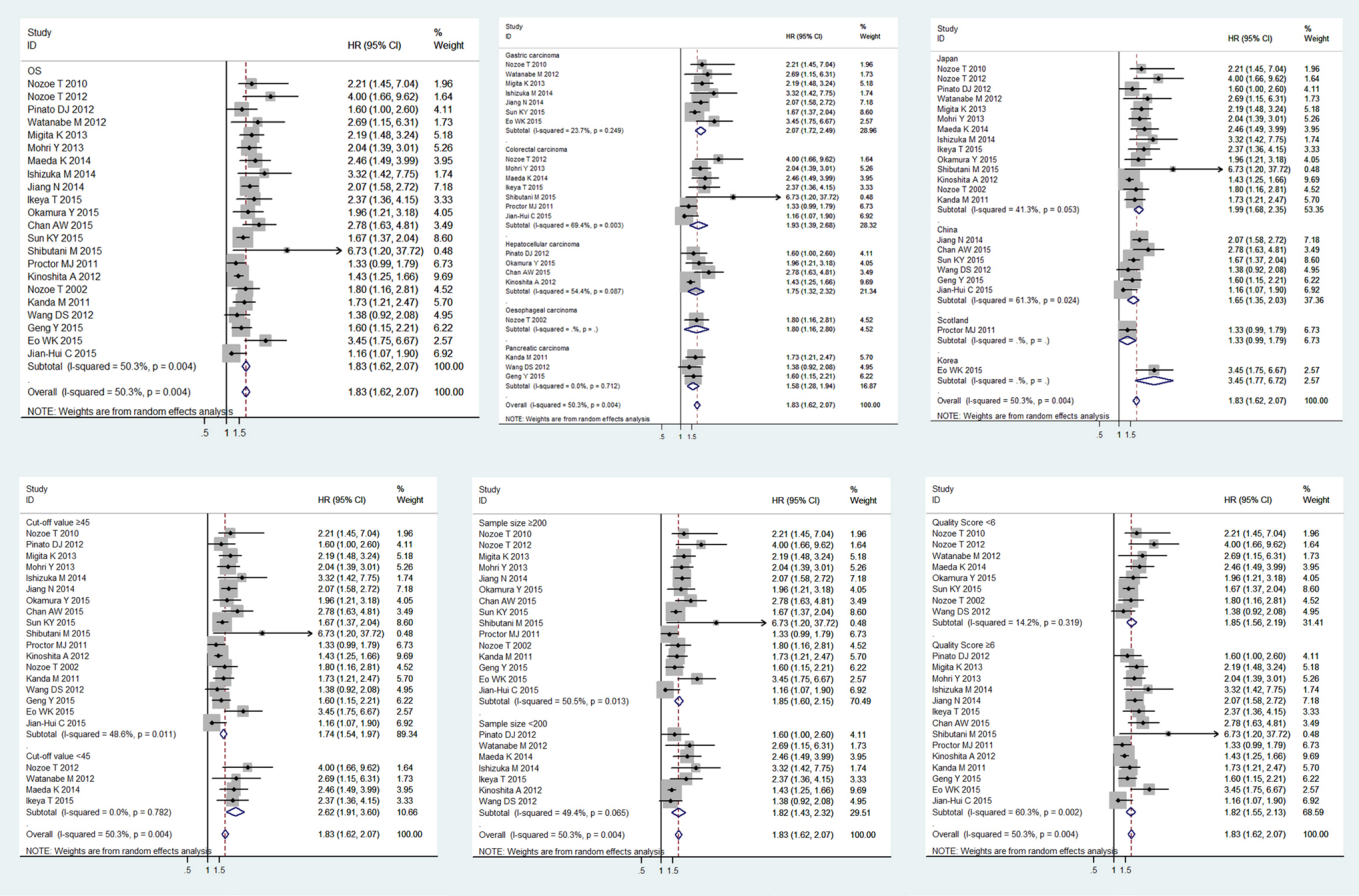
Meta-analysis of the associations between the PNI and OS The segments represent the 95% CIs of each study. The diamonds represent the overall effect sizes, and the diamond widths represent the overall 95% CIs. (**A**) OS; (**B**) tumor type; (**C**) region; (**D**) cut-off value; (**E**) sample size; (**F**) quality score.

The relationship between PNI and OS in patients with digestive system carcinomas was explored in 22 studies with a total of 7,009 enrolled patients [4–7, 9, 16–18, 20–33]. We observed a positive association between PNI and OS in patients (pooled HR 1.83, 95% CI 1.62–2.07), indicating that lower PNI was associated with shorter OS (Figure 2A). However, the heterogeneity of the study was a bit high (*I*^2^ = 50.3%; *P* = 0.004). Then, we stratified the studies into subgroups according to human cancer type, sample size, region, cut-off value, and paper quality score to explore the differences between the subgroups in order to elaborate the prognostic potential of PNI for OS (Table 2). Among five types of carcinomas, the highest HR was displayed in the patients with gastric carcinoma (pooled HR 2.07, 95% CI 1.72–2.49) followed by colorectal carcinoma (pooled HR 1.93, 95% CI 1.39–2.68), esophageal carcinoma (pooled HR 1.80, 95% CI 1.16–2.80), hepatocellular carcinoma (pooled HR 1.75, 95% CI 1.32–2.32), and pancreatic carcinoma (pooled HR 1.58, 95% CI 1.28–1.94). The heterogeneity had little effect in this subgroup-analysis for the values of *P* > 0.05 (GC *I*^2^ = 23.7%, *P* = 0.249; CC *I*^2^ = 69.4%, *P* = 0.003; HC *I*^2^ = 54.4%, *P* = 0.087; PC *I*^2^ = 0.0%, *P* = 0.712) (Figure 2B).

**Table 2:** Results of subgroup analysis of HR ratio of OS of cancers with low PNI level

Subgroup analysis	No. of studies	No. of patients	Pooled HR [95%CI]	Meta-regression (*P* value)	Heterogeneity I^2^ (%)	Heterogeneity *P* value
Region						
Japan	14	3160	1.99 [1.68, 2.35]	0.000	50.5	0.013
China	6	3161	1.65 [1.35, 2.03]	0.000	61.3	0.024
Korea	1	314	3.45 [1.77, 6.72]	0.000	–	–
Scotland	1	374	1.33 [0.99, 1.79]	0.059	–	–
Sample size						
< 200	7	872	1.82 [1.62, 2.07]	0.000	49.4	0.065
≧ 200	15	6137	1.85 [1.60, 2.15]	0.000	50.5	0.013
Cut-off value						
< 45	4	498	2.62 [1.91, 3.60]	0.000	0.0	0.782
≧ 45	18	6511	1.74 [1.54, 1.97]	0.000	48.6	0.011
Type of cancer						
Gastric cancer	7	2381	2.07 [1.72, 2.49]	0.000	23.7	0.249
Colorectal carcinoma	7	2677	1.93 [1.39, 2.68]	0.000	69.4	0.003
Hepatocellular carcinoma	4	927	1.75 [1.32, 2.32]	0.000	54.4	0.087
Pancreatic cancer	3	766	1.58 [1.28, 1.94]	0.000	0.0	0.712
Esophageal carcinoma	1	258	1.80 [1.16, 2.81]	0.009		
Quality Score (NOS)						
< 6	8	2074	1.85 [1.56, 2.19]	0.000	14.2	0.319
≧ 6	14	4935	1.83 [1.62, 2.07]	0.000	60.3	0.002

For the studies originated from the three Asian countries, the pooled HRs were the following: (Japan: HR 1.99, 95% CI 1.68–2.35; China: HR 1.65, 95% CI 1.35–2.03; Korea: HR 3.45, 95% CI 1.77–6.72). Chinese (*I*^2^ = 61.3%, *P* = 0.024) and Japanese subgroups had significant heterogeneity (*I*^2^ = 41.3%, *P* = 0.053). The one study originated from Scotland demonstrated a similar HR in colorectal carcinoma (HR 1.33, 95% CI 0.99–1.79) [30] (Figure 2C).

The pooled HR was 2.62 (95% CI 1.91–3.60) in the subgroup of studies with cut-off values lower than 45, while the pooled HR was 1.74 (95% CI 1.54–1.97) in the subgroup of studies with cut-off values higher than 45. Latter subgroup (*I*^2^ = 48.6%, *P* = 0.011) displayed significant heterogeneity while the former subgroup didn't (*I*^2^ = 0.0%, *P* = 0.782) (Figure 2D).

**Figure 3:**
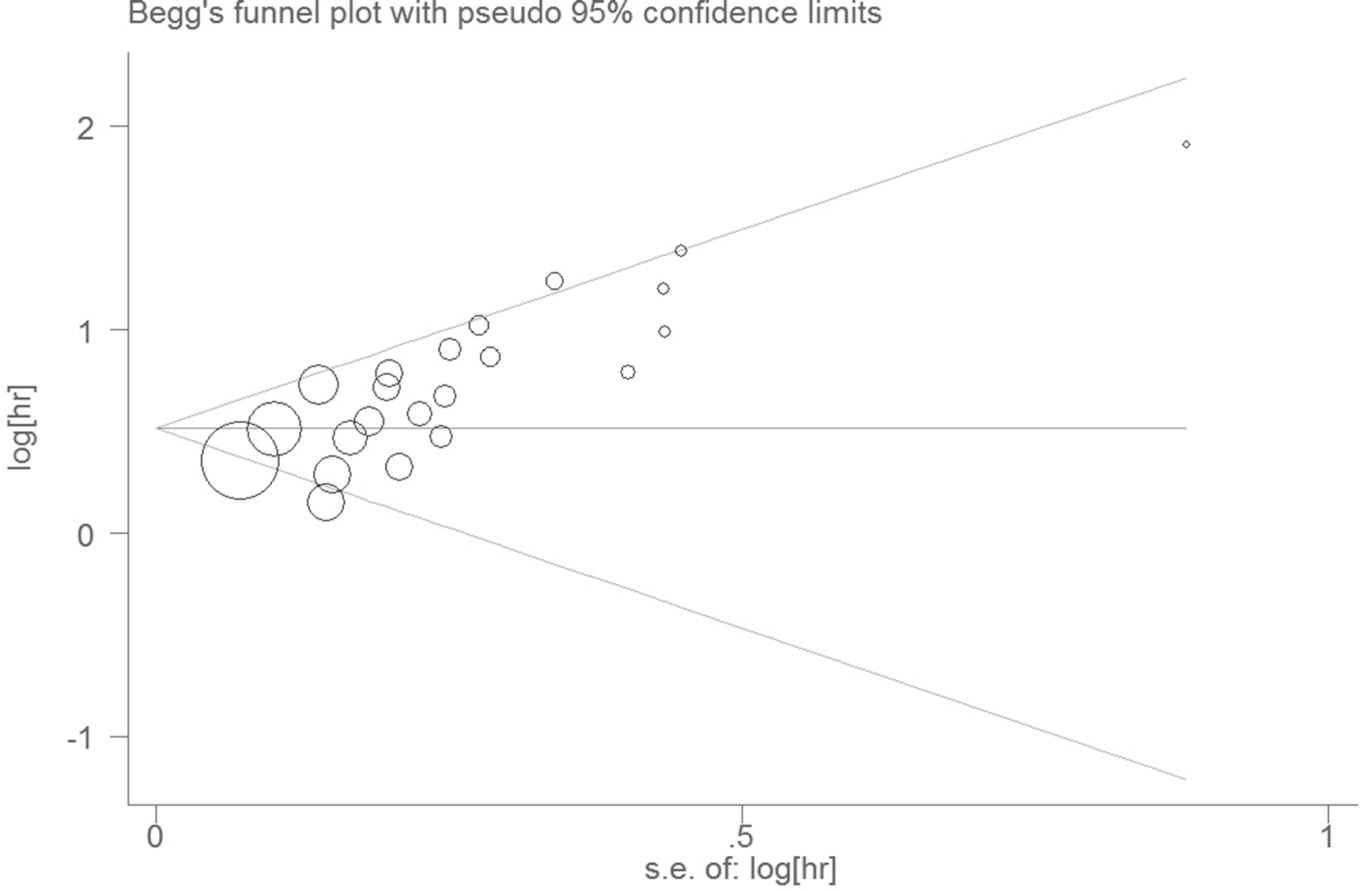
Begg's funnel plot with pseudo 95% confidence limits

**Figure 4:**
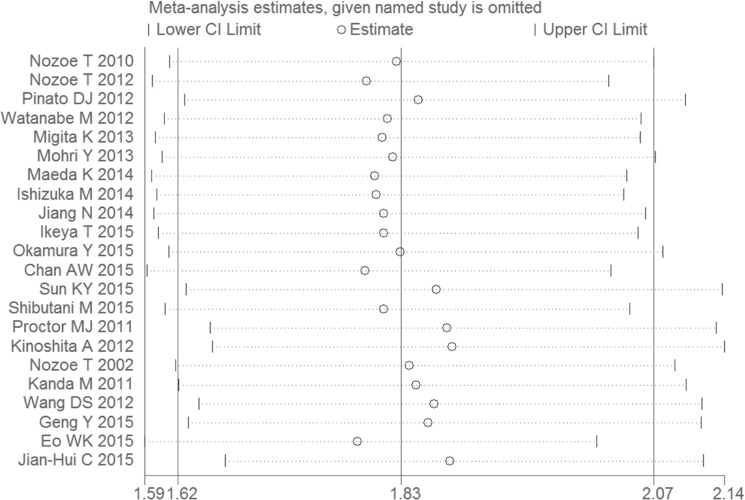
Sensitivity analyses to assess the effect of each study on the overall HR

As taking the sample size and the quality score of the articles into account, the pooled HRs and 95% CIs > 1 were observed in all subgroups (Figure 2E, Figure 2F), the results were consistent with the subgroup analysis. None of the factors mentioned above was responsible for the differences between the subgroups. Publication bias was assessed using Egger's test and Begg's test. Of the two, Begg's test gave a significant *P value* (*P* = 0.001) and the *P value* of Egger's test was 0.000 (Figure 3). To assess the effect of each study on the integrative HR, we conducted a sensitivity analysis. The result showed that the conclusions was not significantly altered after omitting any of the included studies (Figure 4).

### Relationship between PNI and post-operative complications/DFS/CSS

**Figure 5:**
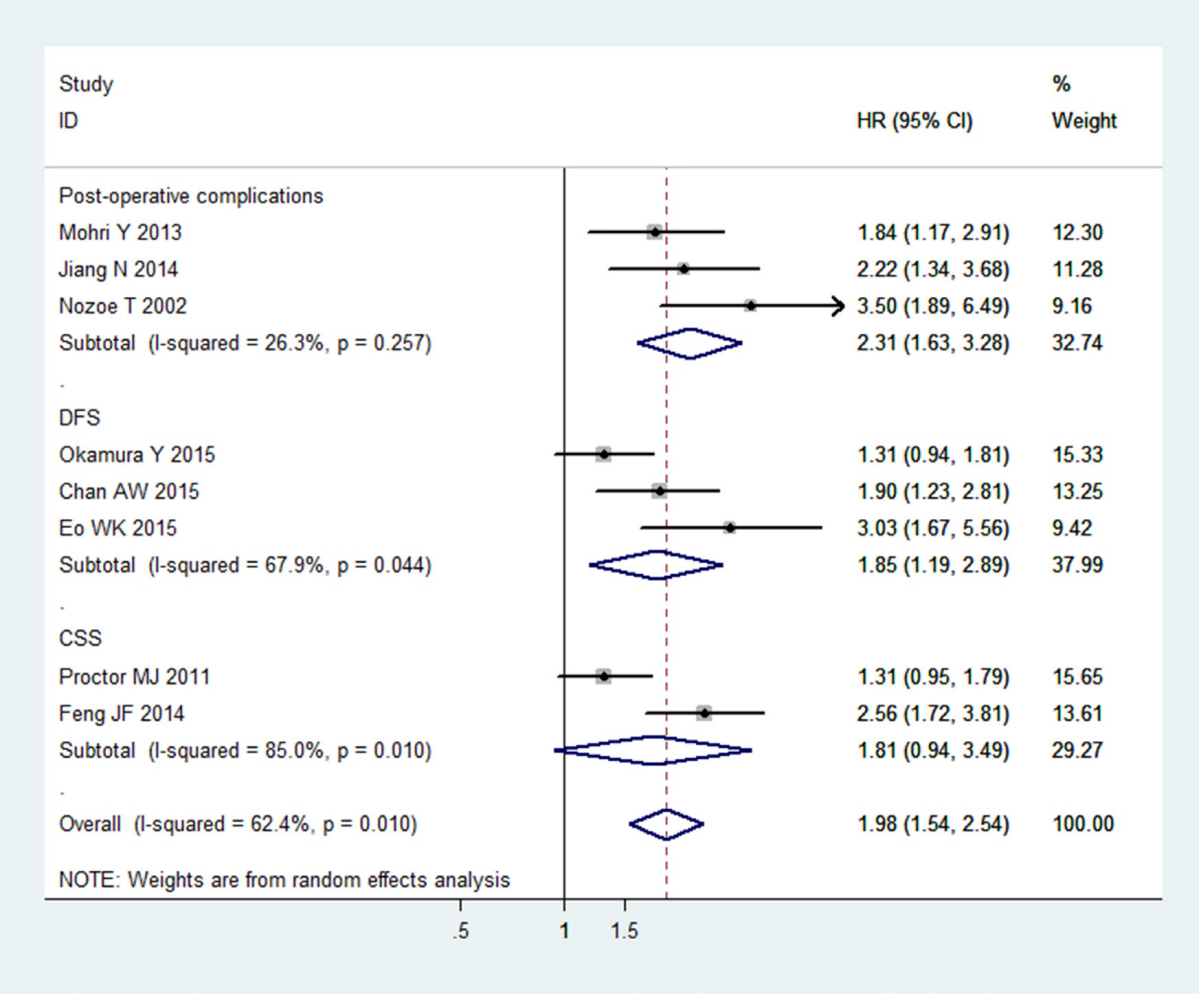
Meta-analysis of the associations between the PNI and post-operative complications, CSS and DFS in cancers The segments represent the 95% CIs of each study. The diamonds represent the overall effect sizes, and the diamond widths represent the overall 95% CIs

There were three studies with a total of 1009 patients, three studies with a total of 979 patients, and two studies with a total of 749 patients analysed the associations between PNI and post-operative complications [5–7], DFS [19, 28, 30] and CSS [4, 18], respectively (Table 3). PNI was defined as an independent predictive factor for post-operative complications (HR 2.31, 95% CI 1.63–3.28), and DFS (HR 1.85, 95% CI 1.19–2.89). The relationship between PNI and CSS was uncertain (HR 1.81, 95% CI 0.94–3.49)(Figure 5). It was noteworthy that the subgroups with regard to DFS and CSS displayed significant heterogeneities (DFS: *I ^2^* = 67.9%, *P* = 0.044; CSS: *I ^2^* = 85.0%, *P* = 0.010). Because of the limited number of included studies, we didn't perform subgroup analysis, meta-regression, sensitivity analysis, Begg's test and Egger's test.

**Table 3:** Results of subgroup analysis of pooled HR of complication, DFS and CSS of cancers with low PNI level

Subgroup analysis	No.of studies	No.of patients	Pooled HR [95%CI]	Meta-regression (*P* value)	Heterogeneity I^2^ (%)	Heterogeneity *P* value
post-operative complications	3	1009	2.31 [1.63, 3.28]	0	26.3	0.257
DFS	3	979	1.85 [1.19, 2.89]	0.006	67.9	0.044
CSS	2	749	1.81 [0.94, 3.49]	0.076	85	0.01

## DISCUSSION

This meta-analysis study aimed to investigate the association between PNI and the prognosis of patients with digestive system carcinomas. 23 studies involving five types of digestive system cancers were enrolled in our meta-analysis. We found that a lower PNI played a negative role as an independent prognostic factor for patients’ survival: shorter OS and DFS, as well as the appearance of post-operative complications of digestive system cancers, especially in gastric carcinomas, colorectal tumours, and hepatocellular cancers. The followings are some possible explanations about the association between low PNI and poor prognosis in cancer patients. Firstly, PNI is decided by the serum albumin concentration and lymphocyte count in the peripheral, both of which are obviously correlated with the prognosis of cancer patients. PNI reflexes the healthy condition of cancer patients. Secondly, poor nutritional status may make patients delay the surgery or adjuvant therapy, even be untreatable for disease. Therefore, this may partly explain the association between low PNI and poor prognosis of patients with cancers. Because of these direct or indirect clues, a low PNI is related to the prognosis of cancer patients.

The Egger’s test and Begg’s test were carried out to assess publication bias. According to the *P* values of two tests, we considered that publication bias existed in this meta-analysis. Although we detected the heterogeneity in the meta-analysis, the sensitivity analysis ensured the stability of the results. There might be several factors being responsible for underlying heterogeneity, such as the patients from different regions, the wide range of publication date, the uneven quality of the papers, the development of detection techniques, the improvement of surgical efficacy and safety, the statistical approach for extrapolating HRs, and the difference in cancer staging criteria. The cut-off value could be considered consistent in our study, but it was still required to verify that 45 was an appropriate cut-off value for our future study. Under these circumstances, individual study may have a considerable impact on publication bias and heterogeneity. Accordingly, more studies with larger sample sizes are required to further confirm this association.

This meta-analysis is the first systematic review concerning about the predictive value of PNI for patients with digestive system carcinomas, and circumvents the limitations of small sample sizes in the individual studies. 23 articles in our analysis included a total of 7,384 participants. Moreover, the data could almost be extracted directly from original articles reducing the approximation bias. Subgroup analysis, Egger’s test, Begg’s test and sensitivity analysis were applied to address this issue. However, there were certain limitations in our study. Firstly, most of the data originated from retrospective observational studies without the use of controls. Secondly, the studies eligible for this meta-analysis were inconsistent with respect to the staging outline of human cancers. Thirdly, all of the included studies almost were Asian origin, so it was unclear whether our findings extended and generalized to other regions of world as well. Thereby, the limitations urged to yield great promise for the addition of more studies with larger sample sizes and more nutritional factors. Moreover, we also sought to link the other nutritional factors with the prognosis of cancer patients, like PLR, NLR and GPS. It was reported that those factors all have negative effects on various cancers patients’ survival [34–42]. Though, these indicators have been studied as simple, inexpensive, and robust prognostic markers in cancers patients many years, it is more likely to improve the accuracy in predicting the prognosis of patients by combination of PNI with other independent prognostic factors.

In conclusion, we demonstrated that PNI was an independent predictive factor for OS, DFS, and post-operative complications. A lower PNI was indeed associated with a poorer prognosis in digestive system carcinomas. It does a favor to determine the optimal timing of surgery or choose the other appropriate individual therapy to adjust patients’ nutritional condition. It also can assist doctors to improve patients’ nutritional conditions by means of early and effective interventions to promote the survival outcomes.

## MATERIALS AND METHODS

### Retrieval strategies

We performed a literature search in PubMed and the ISI Web of Science for articles with the relevance to the association between PNI and cancer patients. The following MeSH and free-text terms were used: ‘prognostic nutritional index’, ‘PNI’, ‘cancer’, ‘carcinoma’, ‘tumour’, and ‘neoplasm’. Subsequently, we checked the references of these papers in order to locate additional articles that could be included in the meta-analysis. We did not exclude articles based on language or publication date. The most recent article had been published on January 12, 2016.

### Selection criteria

After the initial literature search, we handpicked the eligible studies according to the following criteria: (1) the investigations of the prognostic value of PNI in digestive system carcinomas; (2) availability of the necessary data, such as the OR or HR with 95% CIs, or sufficient data to calculate them; (3) availability to the full text; (4) written in English. Abstracts, meetings, and case reports were excluded.

### Assessment of quality

The quality of each paper was assessed using the Newcastle-Ottawa scale. Three aspects were evaluated: selection, comparability, and exposure. A paper can be awarded a maximum of one star for each numbered item within the Selection and Exposure categories. A maximum of two stars can be given for comparability. So, a paper can be awarded 9 stars at most, with more stars indicating higher quality.

### Data extraction

We divided the data that was extracted from each individual study into two categories. The first category contained the characteristics of the studies, including names of authors, publication year, region, tumour type, sample size, age of the patients, cut-off value, treatment, outcome measurements, and quality score. The second category contained the estimated ORs or HRs regarding the prognostic significance of PNI with respect to OS, DFS, CSS, and post-operative complications.

Two methods described in previous studies [43, 44] were applied for the determination of ORs/HRs. The most accurate way was to directly obtain from the original article. When the data were unavailable, ORs/HRs, and 95% CI were calculated based on OR/HR and *P value* or the relative data.

### Statistical analysis

HRs of individual studies were obtained as described above and summarized as pooled HRs. The results presented as HRs and 95% CIs which were first calculated using the random-effects model in order to assess heterogeneity. If heterogeneity was not significant, the fixed-effects model (Mantel–Haenszel) was subsequently introduced [45]. Heterogeneity was determined by forest plots, the inconsistency test (*I*^2^), with *P* < 0.05 indicating significant difference and larger values of *I*^2^ indicating higher heterogeneity. Subgroup and sensitivity analyses were performed to reduce and explain the statistical heterogeneity when that was necessary in order to elaborate on the prognostic role of PNI in cancer patients. Graphical funnel plots were generated for visual inspection in order to qualitatively assess publication bias, while the Begg’s test and the Egger’s test were used for quantitatively determining the extent of publication bias [46, 47]. All calculations for the current meta-analysis were performed using Stata, version 12.0.
